# Tool mastering today – an interdisciplinary perspective

**DOI:** 10.3389/fpsyg.2023.1191792

**Published:** 2023-06-16

**Authors:** Ricarda I. Schubotz, Sonja J. Ebel, Birgit Elsner, Peter H. Weiss, Florentin Wörgötter

**Affiliations:** ^1^Department of Biological Psychology, Institute for Psychology, University of Münster, Münster, Germany; ^2^Human Biology & Primate Cognition, Institute of Biology, Leipzig University, Leipzig, Germany; ^3^Department of Comparative Cultural Psychology, Max Planck Institute for Evolutionary Anthropology, Leipzig, Germany; ^4^Developmental Psychology, Department of Psychology, University of Potsdam, Potsdam, Germany; ^5^Cognitive Neurology, Department of Neurology, University Hospital Cologne, Cologne, Germany; ^6^Institute of Neuroscience and Medicine (INM-3), Forschungszentrum Jülich, Jülich, Germany; ^7^Inst. of Physics 3 and Bernstein Center for Computational Neuroscience, Georg August University Göttingen, Göttingen, Germany

**Keywords:** cognitive foundations of tool use, functional knowledge, object manipulation, problem solving, virtual reality, technological assistants, functional opacity, sensorimotor decoupling

## Abstract

Tools have coined human life, living conditions, and culture. Recognizing the cognitive architecture underlying tool use would allow us to comprehend its evolution, development, and physiological basis. However, the cognitive underpinnings of tool mastering remain little understood in spite of long-time research in neuroscientific, psychological, behavioral and technological fields. Moreover, the recent transition of tool use to the digital domain poses new challenges for explaining the underlying processes. In this interdisciplinary review, we propose three building blocks of tool mastering: (A) perceptual and motor abilities integrate to tool manipulation knowledge, (B) perceptual and cognitive abilities to functional tool knowledge, and (C) motor and cognitive abilities to means-end knowledge about tool use. This framework allows for integrating and structuring research findings and theoretical assumptions regarding the functional architecture of tool mastering via behavior in humans and non-human primates, brain networks, as well as computational and robotic models. An interdisciplinary perspective also helps to identify open questions and to inspire innovative research approaches. The framework can be applied to studies on the transition from classical to modern, non-mechanical tools and from analogue to digital user-tool interactions in virtual reality, which come with increased functional opacity and sensorimotor decoupling between tool user, tool, and target. By working towards an integrative theory on the cognitive architecture of the use of tools and technological assistants, this review aims at stimulating future interdisciplinary research avenues.

## 1. Introduction

Only a few species are using tools (Hunt et al., 2013), and no animal can compare to modern humans concerning the complexity and ubiquity of designing and using tools in everyday life. Knives, pencils, lighters, or power drills: Humans routinely create and employ tools flexibly, they use familiar tools in new situations and new tools in familiar situations (Mizelle and Wheaton, 2010; Vaesen, 2012). Tool mastering relies on a verbatim complex functional architecture because it requires a set of specific sensorimotor as well as cognitive capacities. It can be achieved via different types of learning, and it can be limited in various populations such as non-human primates, human infants, or elderly persons, and it can be impaired in neurological patients with specific tool use deficits such as apraxia or agnosia. Thus, a conjoint interdisciplinary effort is needed to understand the constituents and manifestations of this complex capacity. However, while tool use has fueled research in cognitive neuroscience, developmental and comparative psychology, archaeology, ethology, robotics, neurology, and ergonomics for more than a century, so far there is only little integration between research disciplines, theoretical perspectives, and methods. Hence, tool mastering, i.e., the entire complex of perceiving, selecting, and handling of one or several tools for goal-directed action, remains little understood. In modern times, technical innovations, allowing for remote, virtual, or digital user-tool interactions, have added to the complexity of this topic.

The present paper presents a conceptual framework to study tool mastering in an interdisciplinary manner by focusing on the integration of its sensory, motor, and cognitive underpinnings. With this framework in mind, we will provide a comprehensive review of current evidence from several disciplines to tackle some prevailing fundamental research questions: How, and due to which circumstances, has tool mastering evolved during human phylogeny, and how does it develop in the individual during ontogeny? How are tool perception, selection, and motor handling organized in the brain, and how and why can these processes be selectively impaired in certain neurological patients? Which particular faculties are required for mastering classical mechanical and modern tools, for using them in the real world or in VR, and to what extent is the use of technological assistants comparable to tool use? Which insights in the cognitive architecture come from robots or computational models that learn to recognize, select, and purposefully use tools? In-depth research has revealed that answers to these questions are anything but simple. The here proposed framework will help to structure the existent empirical findings, reveal research gaps and potential connecting points of the disciplines, and thus inspire new research. Finally, the framework will be adapted to the challenge of explaining the mastering of non-mechanical instruments, including the usage of digital devices and virtual tools as well as technological assistants, with the key question of how this relates to the mastering of “good old” mechanical tools.

## 2. Defining tools and tool use

To study tool mastering in an interdisciplinary manner, it is crucial to begin with a scientifically satisfying, comprehensive, and functional tool (use) definition that is neither too narrow nor too broad.

This step turns out to be complicated by the fact that humans excel in constantly inventing new tools. In particular, this journey of innovations features several leaps above and beyond simple (mechanical) tools, which may have fundamental implications for the question of what a tool is: the advent of (a) information-gathering tools, (b) externally actuated (e.g., electrified) tools, (c) virtual reality (VR) tools, and (d) computing tools and technological assistants. In the following, we will provide a basic definition for tool (use) before looking at (a) to (d) tools for examining the modifications of the definition that this would entail. Two extreme cases are conceivable: Either one or several of these special types of tools simply overstretch the conventional definition, and therefore they cannot be considered tools in any proper sense (instead, some other concept would be more appropriate for them); or we need to modify the definition of tool (use) to take them, too, into account. We will argue in favor of the former alternative, showing that a classical definition is able to incorporate some non-mechanical tools, and avoid the latter, as the tool (use) definition would become too broad to be scientifically useful. The goal of these discussions is to offer an analysis that dissects some obvious conceptual or logical needs and scientific consequences of adapting the tool definition that is currently mostly in use.

We propose that five conditions, modified from Shumaker et al. (2011), can be considered mandatory for objects to qualify as *tool*, and for behaviors as *tool use*.

1. A tool is an object that can be manipulated.
2. During tool use, the tool is touched, held, or otherwise directly manipulated by the tool user.
3. The tool is used in an appropriate context, namely purposefully to achieve a goal.
4. The tool is used to alter the state of another object (including other organisms and the tool user).
5. The tool extends the user’s abilities to achieve certain goals.

Now, how does this definition cope with (a) information-gathering tools, (b) electrified tools, (c) VR tools, and (d) computing tools and technological assistants?

Regarding type (a) tools that are made and used for gathering information, for instance, yardsticks, these are covered by the given definition when suggesting that tools include objects that are used to alter the sensory input or the knowledge of the user or of another organism (*cf.* Shumaker et al., 2011, p. 10). For instance, if I measure how tall my dog is, using the yardstick changes *my knowledge* of my dog’s size. Likewise, if I use my telephone to persuade my grandpa to go to the doctor, using the telephone may change my grandpa’s *attitudes or plans*. From the materialist’s point of view, this is not a problem, because mental states are physical states, so if we use the term “state” in this sense, we tacitly include mental states. Even if one accepts this reading without further ado, one must be aware that extending the definition to include mental states means accepting that some effects of tool use are not *directly observable* – which is quite in contrast to how we usually think of tool use.

Type (b) tools like drilling machines, jigsaws, or lawnmowers save an incredible amount of time and give us superhuman strength. Thus, the development of electrified or otherwise externally actuated instruments has extremely expanded our possibilities, but integration into the given definition is still possible without a problem. For instance, when you use a hairdryer, you change the physical state of your hair from wet to dry (so Condition 4 is met). In addition, the hairdryer extends your abilities to achieve this goal in the sense that you are much quicker than when rubbing your hair dry with a towel (fulfilling Condition 5). With regard to Condition 1, the use of type (b) tools often entails different movements than those of a mechanical tool with the same function. Compare, for example, the movements involved in guiding an electric drill compared to using a mechanical drill. However, and this seems particularly important to us, type (b) tools stay under full manual control during usage.

Virtual tools (c) refer to tools that are simulated in VR. Virtual tools are based on generic placeholder objects and have to be distinguished from other kinds of VR interfaces like data gloves employed to engage in VR action. Applying the given definition, VR tools qualify as tools if Condition 1 is meant to include *simulated (virtual)* objects, which in contrast to real objects only the tool user can see. In contrast to type (d) tools, as we will point out, Condition 1 is fully met by virtual tool use since VR hand controllers, i.e., *placeholder objects,* are grasped and moved by the hands and they remain under full control during manipulation. Accordingly, Condition 2 must be read such that touching, holding, or manipulating can only refer to the placeholder object, and finally, Condition 4 has to refer to state changes of simulated (virtual) objects since actions with virtual tools occur in VR.

The given definition, however, appears challenged by computing tools (d) like smartphones or smartwatches, i.e., computers that you can hold in your hand or wear on your wrist, or technological assistants that you control verbally or remotely. In particular, computing tools meet the definition only when we accept a certain reading of Condition 4 (alteration of state), and they have inherited this problem from type (a) tools for gathering and transmitting information, which could be considered to be their ancestors. Just like these, using such a computer causes an overt or covert change of state in the user. For the usefulness of the above definition, however, Condition 1 turns out to be the most critical: What exactly do we mean by “manipulation” when we consider that computational tools can be controlled by speech or by touching an area on a screen as well? Obviously, this condition is softened into the metaphorical if type (d) tools are to be included. We will return and elaborate on this phenomenon of *sensorimotor (de) coupling* in Section 5.

At this point, it is important to understand that robots, which one could consider as the most advanced computational tools to date, are used on the one hand as sophisticated technological assistants or substitutes for humans, and on the other hand as a model for human cognitive processes (including human tool use). The former could, for example take the shape of a robot that performs certain sub-action in an industrial assembly process (helping a human) or even a machine that does this assembly all on its own; the latter could be – for example – a humanoid robot like ARMAR (Asfour et al., 2006) that learns to use tools, e.g., in the kitchen. In this dual role – assistant versus autonomous cognitive model – our approach also considers robotic contributions to understanding natural tool use by living beings.

To summarize, the given tool (use) definition can be read to include *modern tools*: information-gathering tools, electrified tools, and virtual tools. But it is overstretched when it comes to computing or type (d) tools.

Based on the conceptual framework that we provide in the following, we will be able to more accurately determine the differences between classical tools and modern tools, and outline specific research questions addressing this issue. Before we come to this, another critical point remains to be addressed: Regarding Condition 3, the premise of purposefulness (i.e., goal-directedness) is fundamental, yet not easy to prove, because individuals can inadvertently bring about distal effects by tool manipulation, without having strived for this action goal (Seed and Byrne, 2010; Proffitt et al., 2023). Such “accidental” outcomes do not qualify as tool use. Purposefulness also involves psychological agency which means that an organism moves flexibly towards a goal (in this case: involving tool use), even if the context is unknown. It can choose between several possible actions and selects the appropriate one (c.f. Tomasello, 2022; here: the appropriate tool). Thus, as clear indicators of purposeful, goal-directed tool use, two observable and testable behavioral hallmarks have been proposed (Seed and Byrne, 2010): *Selectivity* means that a tool is selected and/or adapted for a particular goal at hand; *flexibility* is evident when different tools are used for the same purpose (Sanz and Morgan, 2009) or when the same tool is used for multiple purposes (Seed and Byrne, 2010; Call, 2013). However, it is not always obvious whether an action with an object is being performed with a goal in mind. Especially in developmental and species-comparative approaches to tool use, but also in robotics, the disposability of these capacities and their necessity for defining a behavior as goal-directed tool use are topics of investigation (Fragaszy and Mangalam, 2018; Colbourne et al., 2021).

## 3. A conceptual framework of tool mastering

**Figure 1:**
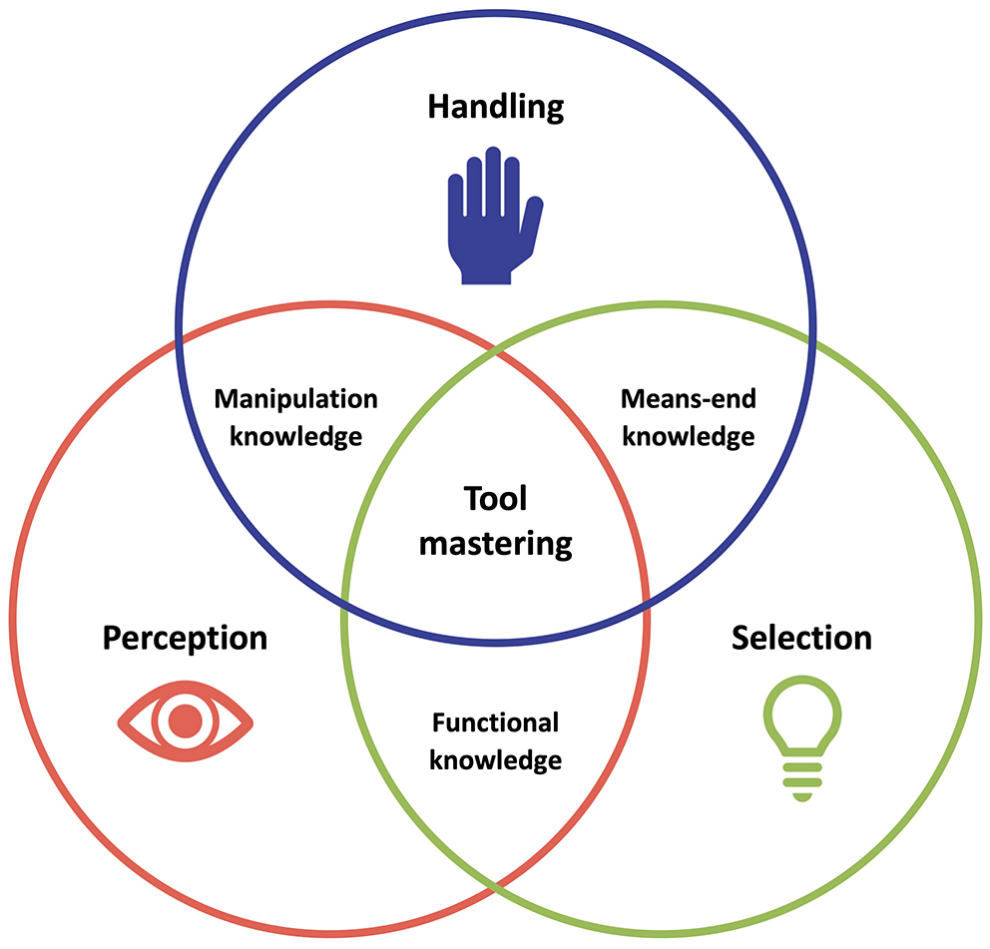
Tool mastering as three basic faculties and their intersections. Tool mastering (ABC) is defined as the interplay of tool perception (A), selection (B), and handling (C). The intersection of perception and handling integrates to manipulation knowledge (AC), the one of perception and selection to functional knowledge (AB), and the one of selection and handling to means-end knowledge (BC).

The complex function of tool mastering includes three basic faculties and their intersections (Figure 1; van Elk et al., 2014 for a somewhat different proposition on action semantics).

Tool mastering calls for registering and analyzing perceptual features of a tool with respect to the given context (tool perception, A). Furthermore, it requires cognitive skills that allow an agent to select the functionally appropriate tool (s) and to plan their use to attain certain action goals (tool selection, B). Finally, motor skills for appropriate tool-related movements (tool handling, C) are needed. We suggest that tool mastering cannot be understood from any of these basic faculties alone. Rather, progress may emerge from focusing research on the intersections of tool perception, selection, and handling, which are: manipulation knowledge (AC) relating tool perception and handling, functional knowledge (AB) relating tool perception and selection, means-end knowledge (BC) relating tool selection and handling, and finally, *tool mastering* (ABC) relating perception, selection, and handling (Figure 1).

These basic faculties and their intersections can be considered on different timescales. On a *temporal microscale,* manipulation knowledge, functional knowledge, and means-end knowledge have to be coordinated and concerted instantaneously in each situation that entails planning and executing purposeful and goal-directed tool use.

On a *temporal macroscale,* the basic perceptual, motor, and cognitive faculties and their interactions have evolved in various phylogenetic specifications, and they change over the lifetime due to learning-induced dynamic plasticity, developmental maturation, normal aging, or neuropsychological pathologies. Investigating the three major building blocks of tool mastering on these timescales can help to generate a comprehensive, integrated theoretical model and allows formulating a set of important and innovative research questions concerning classical and modern tool mastering.

**Figure 2:**
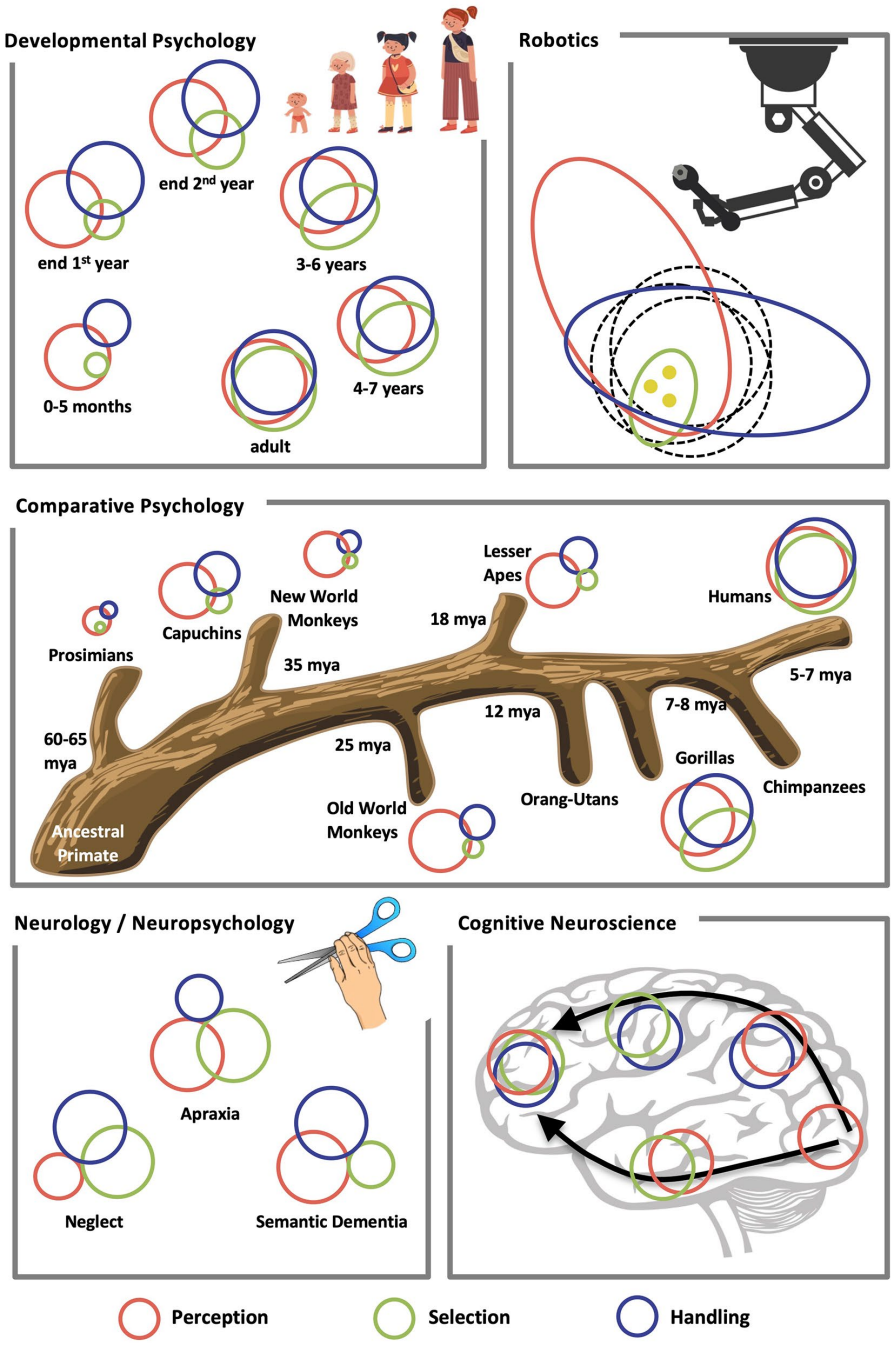
Tool mastering in different disciplines. Tool perception (A, red [medium grey]), selection (B, green [light grey]), and handling (C, blue [dark grey]) are illustrated in the context of different disciplines. “Bubbles” symbolize skill level (bubble diameter) and level of integration (bubble overlap). Ontogenetic development of tool mastering entails advancement in perceptual, motor, and cognitive skills, and also increasing functional, manipulation, and means-ends knowledge. In evolutionary terms, species are equipped with different levels of the basic faculties required for tool mastering, with only the ape species and capuchin monkeys becoming proficient tool users [plus a few Old World monkey species, namely some species of baboons and macaques, c.f. Colbourne et al. (2021)]. A speculative structure for the primates is shown here, as the data are not yet conclusive in some cases. Different neuropsychological pathologies can be depicted by distinctive reductions in size and overlap of the affected basic faculties. From a cognitive neuroscience perspective, integration between tool perception, motor handling, and cognitive selection presumably increases along the ventral and dorsal pathways towards the frontal lobes. In robots, tool-related sensory and motor capabilities can exceed and be different from those of humans (dashed), tool selection is quite underdeveloped, and tool mastering is only pointwise possible ([yellow] dots).

In this review, we will focus on some of the key disciplines in tool use research: The disciplines of developmental and comparative psychology, cognitive neuroscience, neurology, and robotics have so far substantially contributed to our current body of knowledge about tool mastering (Figure 2). In the field of comparative psychology we will concentrate on primates and exclude other animal species (Shumaker et al., 2011; Colbourne et al., 2021), because we set as focus the human being and the human evolution. In the following, a summary of these achievements will be structured according to the three major building blocks of tool mastering.

The distinction between tool-related functional knowledge and tool-related manipulation knowledge (*cf.* AB and AC in Figure 1) is a common thread in many research disciplines, including experimental psychology (Osiurak and Badets, 2016), neuropsychology (Buxbaum et al., 2000), cognitive neuroscience (Boronat et al., 2005), developmental psychology (Kahrs and Lockman, 2014), comparative psychology (Manrique et al., 2010) and computational neuroscience/robotics (Sahin et al., 2007). Functional and manipulation knowledge is also reflected in the basic concept of *affordance*, which first expresses in general terms a specific relation between an object and the actions applied to this object. Originally introduced by Gibson (1979), this concept still pervades tool use research in animals, humans, and robots (Jeannerod, 1994; Stoytchev, 2008). For decades, segregated processing pathways of the brain were suggested for object perception (called “ventral stream”) and object-related action (called “dorsal stream”; Ungerleider and Mishkin, 1982). Meanwhile, we know that this segregated dual-pathway processing is complemented by interactive information exchange (Mahon et al., 2007; Almeida et al., 2010; Bracci et al., 2016; Kleineberg et al., 2018). Moreover, further specifications of the dorsal stream have been put forward in line with multiple parallel parieto-premotor circuits in the macaque and human brain, among which the so-called ventro-dorsal route (i.e., a ventral sub-branch of the dorsal route) which is presumably tailored to exploiting affordances for tool use (Binkofski and Buxbaum, 2013). Furthermore, in robotics (Jamone et al., 2018; Mar et al., 2018), the concept of affordance has been extended by so-called Object-Action-Complexes (Wörgötter et al., 2009; Krüger et al., 2011). These conceptualize objects and tools as not only “suggesting” actions in a Gibsonian sense, but also assume that action plans trigger an active search for those objects and tools that instantiate the required action affordances.

Notwithstanding its ongoing popularity, the notion of affordance has become an issue in itself as researchers have started to take into account that objects (or environments in general) imply not only a set of physically possible actions (according to Gibson’s original formula) but also the subset of actions that we have learned to associate with these objects (or environments). For instance, Gibsonian affordance is meant when describing a stone that can be used for hammering just because it has a certain weight, stability, and size. In addition to this, affordance in the sense of being associated with learned actions is meant for instance when describing an eraser being used for erasing because we have learned that erasers can be employed to erase something written or drawn with a pencil. Thus, an object’s affordance is brought about not only by its raw perceptible features, but also by cognitive processes exploiting our experiences with this object (*cf.* Figure 1: intersections AB and BC). Moreover, humans habitually reason about objects as being designed for a specific function, indicating that tools are usually embedded in a social environment (Hernik and Csibra, 2009; Ruiz and Santos, 2013; Osiurak and Reynaud, 2020). Reflecting on these perceptually-driven bottom-up versus experience-driven top-down sides of tool affordance inspires novel research questions on perceptual and motor learning as well as in cognitive learning of tool use during human ontogeny and phylogeny as well as in robots, as will be outlined below.

## 4. Three building blocks of tool mastering from various disciplines

### 4.1. Tool manipulation knowledge

Tool manipulation knowledge connects tool perception and tool-related motor skills. The foundations for using tools are laid from early on. By touching, mouthing, or banging objects, infants gain tool-related sensorimotor experiences, thereby exploring perceptual object features and action possibilities. Purposeful and skillful use of familiar tools (e.g., spoons) then begins at about 8 months (Greif and Needham, 2011) when infants also start imitating object-directed behavior. With the advancement of fine-motor skills and sensorimotor coordination, manipulation knowledge increases during the following years through own object exploration and observing other people’s tool use (Elsner, 2007, 2009; Needham, 2016). One important research issue is how different types of learning (see below), which emerge during early childhood, promote progress in tool mastering. Statistical learning, i.e., representing the temporal/spatial contiguity or probabilistic contingency of sensory and/or motor events, and reinforcement learning based on classical and operant conditioning are present already during the first months of life (Adolph and Berger, 2011; Needham, 2016). Around the first birthday, trial-and-error learning kicks in, due to advanced motor and behavioral control, and imitation and observational learning informs the infant about how others handle tools, and which action effects usually follow from that (Elsner, 2007). In the following years, enhanced higher-level cognitive processes and functional tool knowledge as well as procedural knowledge allow for increasingly controlled, planned, and skilled tool manipulation (Keen, 2011). However, the exact developmental course of the basic faculties of tool mastering and their age-related intersections across the lifespan are far from being understood (Yoo et al., 2016), and studies on children’s mastering of modern tools are still rare (Dejonckheere et al., 2014; Cristia and Seidl, 2015).

Primate species have adapted to their ecological niches concerning the frequency and complexity of object manipulations: The infant’s transition from explorative object manipulation to purposeful tool use is mirrored in the increasing integration of object perception and handling (Heldstab et al., 2020) from New World monkeys (except capuchin monkeys) and lemurs, to Old World monkeys (except leaf-eaters), to finally capuchin monkeys and apes (Glickman and Sroges, 1968; Torigoe, 1985). Notably, only a few species (e.g., chimpanzees, Sumatran orangutans, capuchin monkeys) have developed habitual tool use for extractive foraging in the wild, with some species showing cultural variability across populations (Whiten et al., 1999; Koops et al., 2014). However, an impact of environmental or learning factors is indicated by the finding that all great ape species use tools in captive settings, as do some monkey species (Tomasello and Call, 1997; Colbourne et al., 2021). Further research is needed here to distinguish between genetic, environmental, and learning factors enabling goal-directed tool use in primates and human children (Meulman et al., 2013). As in human children, the perception and handling of objects become more integrated during ape ontogeny (Poti and Spinozzi, 1994; Biro et al., 2006; Hayashi et al., 2006), with complex bimanual tool use such as nut-cracking in chimpanzees being first successfully performed rather late during ontogeny, namely by 3 to 3.5 years of age. Interestingly, young children exhibit a dissociation between tool perception and tool handling, in that they show some understanding (e.g., for causal relations) before they can act on objects (Bonawitz et al., 2010; Greif and Needham, 2011), but it is unclear whether such dissociation also occurs in (non-tool-using) primate species.

As mentioned above, object explorations give insights into the manipulation possibilities of objects based on perceptual feedback. An interesting aspect of ape tool use is the intrinsic motivation to explore objects, which can foster future tool use (Call, 2013; Ebel and Call, 2018). However, apes have not yet been shown to actively seek explanations or test hypotheses (e.g., for causal structures) with their explorations in absence of extrinsic rewards, as can be observed in human children (Schulz and Bonawitz, 2007), an exciting field for future research. Humans have evolved highly refined verbal and socio-communicative skills that are essential for imitative learning, social instruction, and cultural transmission of tool use (Morgan et al., 2015; Stout, 2018). If language and teaching have potentially co-evolved with tool use during human phylogeny, how does tool use in primates relate to that? It has been shown that chimpanzees exhibit emulation and imitation learning (but not necessarily teaching), communicate flexibly with gestures (and perhaps even vocalizations), and pass on tool use to the next generation (Whiten et al., 1999; Horner and Whiten, 2005; Pika et al., 2005; Slocombe et al., 2022). A current research topic is compositionality in their gestures and vocalizations and how this is linked to tool use (Steele et al., 2012; Amici et al., 2022; Girard-Buttoz et al., 2022). How communicative skills and teaching have thus played a role in the evolution of (human) tool use is something that future research will show (Steele et al., 2012).

Interestingly, very similar dissociations between tool perception and tool handling can be observed in neurological patients: Object perception is impaired in agnosia due to ventral stream lesions, whereas tool handling is impaired in apraxia due to dorsal stream lesions (Coslett, 2018). And yet, this functional dissociation does not reflect an absolute dissociated neural organization of tool perception and tool handling capabilities. Rather, several functionally specialized parietal-premotor loops work in concert to relate pragmatic object properties such as shape, orientation, or location to appropriate hand shapes for grasping and manipulation movements (Fagg and Arbib, 1998; Chen et al., 2015). Thus, the so-called ventro-dorsal route (including anterior intraparietal area AIP, posterior temporal and ventral premotor cortex) stores abstract, skilled multimodal manipulation knowledge, while the so-called dorso-dorsal route (including parietal area V6a and dorsal premotor cortex) encodes object shape, orientation, and location to serve online fine-tuning of tool use, including reaching, based on current visual and somatosensory input (Rizzolatti and Matelli, 2003; Buxbaum, 2017). The ventro-dorsal route was also proposed to constitute a complex interface between the mostly action-related dorsal stream and the rather perception-related ventral stream (Grafton, 2010; Binkofski and Buxbaum, 2013). The notion that close interactions of the two streams are required for complex functions such as tool mastering (Milner, 2017) is also supported by findings in patients with tool use deficits. For instance, in semantic dementia affecting ventral stream functions, a degraded conceptual information about tools is sometimes also associated with impaired manipulation knowledge (Hodges et al., 1999, 2000). In the same vein, in apraxia following dorsal-stream lesions, the deficits in tool manipulation can come with degraded functional tool knowledge (Martin et al., 2016).

These clinical and above-mentioned developmental findings suggest that tool mastering should be reconsidered in the light of new evidence for specific interactions between the ventral and dorsal stream and their maturation (Kersey et al., 2015). Also, studies in patients with circumscribed lesions of the inferior parietal lobule, a hub in the ventro-dorsal stream which is supposed to act as an interface between the two streams, will be of particular interest. Additionally, investigating the learning of using novel tools or of substituting tools could test the potential dynamic learning trajectory from dorsal-to-ventral representations (Tobia and Madan, 2017). This in turn could be flanked by testing whether patients with lesions in either stream can learn the use of novel tools or, more generally, selective and flexible tool use.

The question of how to efficiently identify and code tool-related sensory and motor information is relevant not only to neurocognitive studies, but also when constructing robots. Currently, in robotics visual data are represented either explicitly (e.g., by a histogram of oriented gradients or other explicit image features) or implicitly by the weights of a deep neural network. To encode motor information, splines, Dynamic Movement Primitives (Schaal et al., 2005), and Gaussian Mixture Models (Calinon et al., 2007) are often used. However, since efficient robots need to show some degree of autonomy, the crucial question became how to represent sensory and motor data so that they seamlessly merge with cognitive autonomous processes. Understanding the interplay of the neural processes of sensorimotor tool mastering discussed above may offer a pathway towards this. However, research will also benefit from a scientific exchange in the other direction, because robotics has enabled a deep understanding of the enormous complexity of the required sensorimotor models. Point-wise transfer of this model-knowledge into the life sciences is sometimes visible, for example when making models of action processing (Giese and Rizzolatti, 2015).

### 4.2. Functional tool knowledge

Functional tool knowledge relates the individual’s intentions and action goals to the perception of the functional properties of tools that may be suitable for achieving those goals. This process goes beyond simple sensorimotor activation, as tool selection requires the ability to form intentions and the motivation to translate those intentions into overt action (Lockman and Kahrs, 2017). This is reflected in the prolonged ontogenetic trajectory of effectively selecting classical tools, which is well developed only in 3- to 6-year-old children (Keen, 2011). While already 4- to 8-month-old infants categorize objects according to physical features such as shape or rigidity (indicating sensorimotor processing of potential tool-properties), the second year of life comes with a shift to sorting by demonstrated functions, indicating processing of objects’ potential to induce changes in the environment when being manipulated (Poulin-Dubois and Pauen, 2017). Fittingly, imitation and observational learning improve infants’ tool use from about the first birthday (Elsner, 2009). This is supported by social instruction, scaffolding, and cultural transmission of tool use during the following years, based on development of the required verbal and socio-cognitive abilities, such as the understanding that others’ object-related actions are based on internal goals and intentions (Tomasello et al., 2005). While successful tool manipulation can also result from trial-and-error learning without functional tool knowledge, children need to develop some causal understanding of the relation between objects and goals or desired effects for insightful tool mastering (Keen, 2011). Thus, across the first years of life, humans become increasingly able to not only relate perceptual object features to object handling (i.e., tool manipulation knowledge) but also to use tools in the way for which they were designed (Rachwani et al., 2020). Future investigations need to elucidate how developmental advancements in attention, memory, self-regulation, language, socio-cognitive capacities, and types of learning (and their dynamic interplay) contribute to young children’s expanding functional tool knowledge (Lockman and Kahrs, 2017).

So far, we know that all great ape species and some monkey species can select tools based on perceptual functional properties (e.g., contact, rigidity; Hauser et al., 1999; Manrique et al., 2010). For example, great apes select novel tools based on their perceptual rigidity to obtain a food reward on the first trial; thus, there is no requirement for learning about how to handle the tools, yet handling or observed handling increases success rates (Manrique et al., 2010). Interestingly, only great apes show some understanding of the causal relations of the use of the tool and the elicited effects (Hanus and Call, 2011; Völter et al., 2016). Moreover, it is still under discussion whether non-human primates have enduring functional object representations (i.e., tools represented as being for specific purposes), as humans have (Vaesen, 2012; Ruiz and Santos, 2013). Although great apes show relatively high degrees of innovation and flexibility in their tool use (Manrique et al., 2013; Motes-Rodrigo and Tennie, 2022), they may get inflexibly fixated on familiar tool functions, resulting in a decrease in problem-solving performance when required to apply a new function to a given tool (Gruber, 2016; Ebel et al., 2021). Whether this *fixedness* is based on functional representations or sensorimotor processes (and then it would rather fall under means-end knowledge, see next section) need to be examined more closely as well as the interaction of both processes (flexibility and fixation), also considering similar phenomena in human children’s tool use (German and Defeyter, 2000; Defeyter and German, 2003).

One factor contributing to the ontogenetic and phylogenetic phase-lag of tool-selection abilities relative to tool-handling skills may be the remarkably prolonged maturation of heteromodal association cortices, especially in the temporal lobe. Tool selection based on semantic functional tool knowledge relies on the posterior middle temporal gyrus as well as the inferior and anterior temporal cortex of the ventral pathway (Chen et al., 2015; Lambon Ralph et al., 2017), and grey matter of the temporal cortex (except for the temporal poles) continues to mature after the grey matter of other association cortices (Gogtay et al., 2004). Despite the undisputed central role of the temporal lobe for semantic processing, including object and tool meaning, it is now discussed whether the anterior temporal lobe, or rather the posterior middle temporal gyrus, qualify as the “semantic hub” in the widespread semantic brain network (van Elk et al., 2014; Lambon Ralph et al., 2017). In support of at least partly independent neural substrates, semantic dementia, related to dysfunctions of the temporal cortices (ventral stream), mainly affects functional tool knowledge (Hodges et al., 2000), whereas apraxia, related to dysfunctions of the inferior parietal cortex (dorsal stream), mainly affects manipulation knowledge (Buxbaum and Saffran, 2002; Goldenberg and Spatt, 2009). However, functional and manipulation knowledge can be affected to varying degrees in both diseases (Hodges et al., 1999), suggesting a complex interplay between their underlying networks as discussed above (Binkofski and Buxbaum, 2013; Milner, 2017; Kleineberg et al., 2018).

As said in the beginning, functional tool knowledge relates the individual’s intentions and action goals to the perception of the functional properties of tools that may be suitable for achieving those goals. Neurocognitive models propose that this interplay between bottom-up perceptual processes and top-down selection and biasing is implemented by ventrolateral prefrontal cortex (vlPFC), a convergence zone of both the ventral and the dorsal stream (Petrides and Pandya, 2009; Takahashi et al., 2013). It is suggested to contribute to tool selection by top-down input to both temporal and parietal tool-related areas. Typically, the vlPFC increases in activity with the capacity of currently available objects to restrict action options (Schubotz et al., 2014; Hrkać et al., 2015; El-Sourani et al., 2018). The top-down signal provided by the vlPFC was proposed serve the selection, and resolve the competition, among semantic alternatives (Badre et al., 2005; Moss et al., 2005), action alternatives (Cisek, 2007; Borghi, 2014; Borghi and Riggio, 2015; Buxbaum, 2017), and/or several concurrently available tools, for instance when organizing several tools in multi-step action (Koechlin and Jubault, 2006). In an interdisciplinary context, *affordance competition* (Cisek, 2007) has been proposed to account for – and was employed to computationally model – deficits in apraxia (Rounis and Humphreys, 2015). Hierarchical affordance competition has also been related to predictive coding (Pezzulo and Cisek, 2016), one of the most influential theories in current cognitive neuroscience *en route* to an integrated model of brain function (Friston, 2005). This connection to predictive coding seems even more attractive because in robotics, functional tool knowledge is commonly associated with the robot’s capability to predict the consequences of its actions (*cf.* ideomotor principle; Shin et al., 2010).

Above, we discussed the complex problem of how to efficiently represent sensory and motor data. Here, we are concerned with *predictive processes* – short-term (behavioral) as well as long-term (learning) – and their algorithmic implementations. Predictions of action consequences can take the form of a typical *forward control model* (Jordan, 1996), where the system tries to predict the sensory signals and events that follow an action. Several algorithms for this have been proposed (Haruno et al., 2001). However, these models exclusively treat signals and do not offer access to more complex – for example explicit (declarative) – action semantics. The latter aspect has been extended by studies that represent the effects of (tool-related) actions by predefined action-categories (Fritz et al., 2006) or probabilistically (Montesano et al., 2007). Action semantics are specifically in the focus of approaches that represent human manipulation actions by using grammar-like structures (Aksoy et al., 2011; Pastra and Aloimonos, 2012). These pre-defined methods are supplemented by studies that use various learning mechanisms to gain functional tool knowledge (Stoytchev, 2008; Mar et al., 2015; Zhu et al., 2015), and by computational modelling of goal-related actions of human adults and infants in probabilistic recurrent networks (Butz, 2016; Gumbsch et al., 2021). Thus, robotics has approached the problem of functional tool knowledge at different semantic levels, for example, to understand the dynamics of tool use by motion trajectory analysis or to make sense of tool-induced situation changes by cause-effect analysis. However, the above-discussed findings of (neuro)-cognitive, developmental, and comparative psychology still need to be reconciled with such a semantic layering approach regarding the processing of goal-related tool-affordances.

### 4.3. Means-end knowledge

Means-end knowledge relates goals to the selection of appropriate tools and tool-using actions. Around the first birthday, infants differentiate between actions (means) and action consequences (ends) in their own and others’ behavior and they show goal-directed use of familiar tools for solving simple problems (Keen, 2011). One-year-olds also begin to master inverse action planning, that is, starting from the intended goal and selecting appropriate tool-handling movements (Deák, 2014). However, more research is needed on the underlying knowledge base (Elsner, 2007) or the types of learning that support goal-related tool mastering (Greif and Needham, 2011; Needham, 2016).

Flexibility, as one of the behavioral hallmarks of purposeful tool use (Seed and Byrne, 2010), is still limited during the preschool years, with perseveration on familiar tool-use handling, limited transfer of familiar strategies to similar problems, and missing alternative plans when familiar strategies fail (Smitsman and Cox, 2008; Elsner and Schellhas, 2012). In observational learning of tool use, preschoolers also tend to over-imitate, performing action steps that are unnecessary to achieve a given goal (Lyons and Keil, 2013). Remarkably, problems with systematic problem solving (including tool-related problems and functional fixedness) continue until adolescence (Defeyter and German, 2003). Therefore, the critical capacities that drive developmental change in tool mastering still need to be characterized, also to better understand the two-step developmental surge in tool mastering, with tool handling preceding tool selection in human ontogeny and possibly phylogeny (Kahrs and Lockman, 2014).

Flexibility in great ape tool use comprises complex action planning, such as sequential tool use (i.e., using a tool to access another tool; Martin-Ordas et al., 2012) or using tool sets (i.e., gathering several tools that are then used with distinct functions; Boesch et al., 2009). It also encompasses substantial delays within the course of an action, i.e., future planning (Mulcahy and Call, 2006). However, the cognitive underpinnings of this behavior and species’ signature limits are not well understood. For example, it is unknown how many steps different primate species can plan ahead and which role experience with the single steps plays. Relating to the latter question, it is discussed whether in patients with so-called *ideational apraxia,* impairments in multi-step actions, involving the selection of appropriate objects, are just an accumulation of deficits at the level of using single tools (De Renzi and Lucchelli, 1988). Other important questions concern the capacity to generalize means-end relations to novel (but structurally similar) problems (Tomasello and Call, 1997; Martin-Ordas and Call, 2009) or the ability for social learning of tool use. When observing tool use of others, human children tend to copy the precise actions (i.e., imitation learning), whereas apes mainly copy the obtained results (i.e., emulation learning) and imitate the precise actions only if the causal structure of the problem is opaque (Horner and Whiten, 2005; Tennie et al., 2010). These observations yield insights into the character (Price et al., 2009; Hopper et al., 2015) and the limitations of primate tool use, such as a potential lack of cumulative culture (Marshall-Pescini and Whiten, 2008; Tennie et al., 2009).

It is a prevailing theoretical motif that means-end relations can be viewed as the fundamental hierarchical structure underlying tool use (Grafton and Hamilton, 2007; Kalénine et al., 2013). However, there is some doubt on a straightforward compatibility between goal-means hierarchies, which are causal control hierarchies, and actions, which are typically described in terms of part-whole hierarchies (Hamilton and Grafton, 2007; Botvinick, 2008; Uithol et al., 2012). In particular, hierarchies ruled by the temporal duration of sub-goals may provide a solution to this conflict (Ondobaka and Bekkering, 2012), but it remains to be empirically tested how this general account translates to the concrete implementation of tool mastering. Thus, tool use may be hierarchically structured, but the levels of this hierarchy are unknown yet (Botvinick and Plaut, 2004; Ondobaka and Bekkering, 2012; Uithol et al., 2012). Here, some striking gaps in tool use research may result from lacking relations to research on executive control of action planning and execution (Koechlin and Summerfield, 2007). Also, hierarchical accounts of tool use need to consider the debate between accounts of *embodied* tool mastering (*cf.* Section 5) favoring a modality-specific, distributed coding of tool abilities, and accounts favoring modality-specific as well as integrated, amodal representations and processes (Patterson et al., 2007). Here, it is vividly discussed whether there is an amodal hub on the top of the tool processing hierarchy, as mentioned above (*cf.* functional tool knowledge), and if so, which brain area or set of brain areas may serve this highest integrative function (Martin et al., 2014; Jung et al., 2017).

Finally, from a robotic perspective, means-end behavior is often linked to *inverse control models*. When transcending mere control signals and their sensory consequences by elaborating complex situations, actions, and situation-changes, inverse models require the modelling of complex outcome-situations or some kind of “mental simulation” (Hesslow, 2002). Other studies tried to combine forward with inverse models to implement tool use in a machine (Schillaci et al., 2012). *Generative modelling* approaches, which use (mental) simulation, are similar to the models of infants’ experience-based tool use described above: Based on some prior experience, which constitutes a model for a certain tool-induced cause-effect relation, the agent will seek to model the effect of the same tool in a different situation or of a different tool in the same situation (Friston, 2005; Butz, 2016). Structural similarities between known and unknown aspects (of the situation and/or tool) are used as the scaffold for this type of generative process (Wörgötter et al., 2015). However, while several general models for tool mastering exist (Wu and Demiris, 2011; Tikhanoff et al., 2013; Rajeswaran et al., 2017), generative models for tool use are still rather feeble. Forward models together with inverse models, applied above and beyond raw control signals and combined with generative processes, appear promising for capturing the complexity of tool mastering, and it is a viable task for the future to substantiate or disprove this claim.

## 5. Modern tools versus technological assistants: increasing functional opacity and sensorimotor decoupling

So far, this review considered research on tools like hammers, knives, keys, etc. – *classical* tools so to say – for which a large body of literature exists. We have suggested that these research findings arise from very different disciplines and would benefit from more cross-linking.

Different from this, there is hardly any research on the use of modern tools. As mentioned in our 5-point definition, traditional concepts of tools and tool use are greatly strained by the fact that modern society has developed tools and ways of using tools that seem to partly decouple the user from the target on the sensorimotor level. Thus, switches, buttons, or verbal commands allow us to control machines; and joysticks, gamepads, and data gloves allow us to modify objects via computer software. Tools for human-machine and human-computer interaction are omnipresent in leisure and work, and still, it is yet unknown whether the underlying functional cognitive architecture and the integration of the involved basic faculties as depicted in Figure 1 persists, or only partly so, when mastering classical and modern tools, be it in reality or VR. In the following, we will re-consider modern tools (and technological assistants) against the backdrop of our conceptual framework.

**Figure 3:**
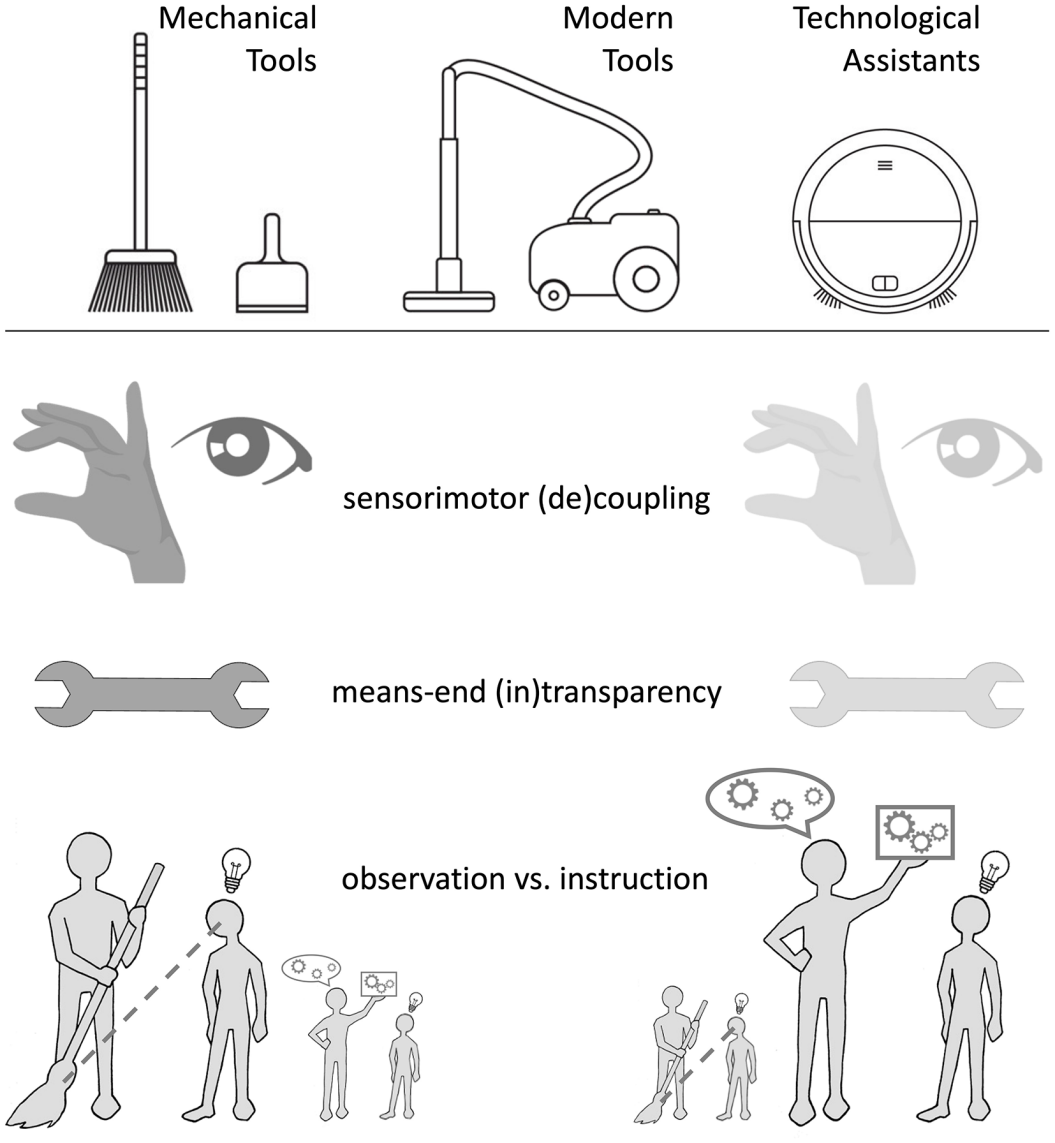
Differences between mechanical tools, modern tools and technological assistants. The use of modern tools, and even more so of technological assistants, differs from the use of classical mechanical tools in the degree of sensorimotor decoupling, the transparency of means-ends relationships, and also in the way we learn to use such tools. Wireless Vectors by Vecteezy (https://www.vecteezy.com/free-vector/wireless).

For classical mechanical tools, the coupling between tool user, tool, and target is *direct* and *(temporally) immediate*. For example, a broom has perceivable features (e.g., bristles) that suggest its selection for a certain purpose (sweeping), and this tool is directly manipulated by the user to purposefully change the state of another object (e.g., dust, the floor). For *modern tools* (e.g., a vacuum cleaner), in contrast, the functional properties as well as the means-ends relations (or cause-effect chains) are far more opaque (e.g., bristles may not be visible). For many *modern tools*, the sensorimotor coupling between tool user, tool and target is far more indirect than for mechanical tools. We think that considering the extent to which sensorimotor coupling is preserved in different modern tools helps a lot in delineating the otherwise rather fuzzy transition between (classical and modern) tools and technological assistants (see Figure 3). For example, when cleaning the floor with a broom, there is undoubtedly a strong coupling between the movements of the tool user and the tool. In the case of electric vacuum cleaners, sensorimotor coupling is still strong, since the device must be moved and fully controlled with force and coordination during the entire process of use. This is not changed by the fact that the movement pattern required by the electric tool is often different from that of the mechanical variant of the same function (here: broom and shovel); see also our comments on this in section 2. Finally, in the case of technological assistants such as vacuum robots, sensorimotor coupling (between the user and the assistant) is eliminated. Since in individual cases the sensorimotor coupling can disappear in stages - a vacuum robot can work on the basis of human remote control, but also autonomously on the basis of artificial intelligence - it can be difficult to distinguish a (very) modern tool from a technical assistant.

Technological assistants, this much seems clear, are not simply comparable to modern tools. Thus, if we look back at our definition from Section 2, it is obvious that Condition 1 and 2 do not apply to technological assistants, while Conditions 3 to 5 apply to them just as they do to modern tools. In the following and final step, we will briefly consider this special class of technological assistants, with particular attention to similarities with and differences from modern tools. Especially, we will focus on the impact of increased *functional opacity* (i.e., means-end intransparency) and *sensorimotor decoupling,* which refer to modern tools and to an even greater extent to technological assistants. For the sake of readability, we will hereafter abbreviate “modern tools and technological assistants” to MTTA where necessary.

We put forward that functional opacity and sensorimotor decoupling (Figure 3) often come with important benefits and facilitation. Many MTTA extend the tool user’s abilities to modify states of objects or organisms to an even greater degree than classical tools do. For instance, surgeons handle smart instruments in a similar way as conventional mechanical surgery tools but achieving much higher precision. Surgical instruments continue to be miniaturized and augmented by robotic control, allowing increasingly sophisticated surgical goals to be achieved with less and less tissue damage. Moreover, motors and built-in mechanics of MTTA allow for goal achievement by simple actions (e.g., a button press), without any need for tool-specific motor skills or deeper insights into how exactly the selected means cause the required ends. For instance, even people with low physical power or limited sensorimotor competencies can mow the lawn with an electric lawn mower or with a robot mower. Also, human primates that do not use classical tools can learn to interact with a touchscreen. Indeed, MTTA can help to successfully transcend many sensory, motor, or cognitive limitations of the tool user, thereby magnifying the capacity to achieve complex tool use goals.

Interestingly, while every effort has been made to improve the functionality of smart modern tools, we are largely ignorant about the specific cognitive capacities that are required for mastering them. In particular, the tool-handling component of manipulation knowledge and means-ends knowledge as well as tool-perception abilities seem to be less critical for mastering modern as compared to classical tools. As outlined below, this has a direct impact on all domains related to the use of tools.

From the neurocognitive perspective, the dorso-dorsal route, which supports on-line fine-tuning of tool use based on current visual and somatosensory input, should be more relevant for classical tools than for MTTA, and more so the greater the sensorimotor decoupling. Neurophysiological studies in macaques and humans have impressively demonstrated that the use of mechanical tools expands the representation of the hand or forelimb: The cerebral body schema is adapted to incorporate the tool (Maravita and Iriki, 2004; Holmes, 2012), while the end-effector (i.e., the locus of control), moves away from the body to the contact between tool and manipulated object or surface, a process called distalization of the end-effector (Arbib et al., 2009). While the assumption that “tool embodiment” is also possible in MTTA needs further empirical corroboration, it is easy to see that the distalization is no longer possible in MTTA due to the abstraction between effector and target.

At the same time, the functional opacity of MTTA and the remarkably increased complexity and sophistication of goals we can aim for when using MTTA often, but not always place greater demands on the functional knowledge, since MTTA rarely display the structural (or functional) properties that – in the case of classical tools - facilitate tool identification (supported by the ventral stream) and goal-based selection (supported by the vlPFC). While a vacuum cleaner (modern tool) still remotely reminds us of a broom, a vacuum cleaner robot (technological assistant) no longer bears any obvious resemblance to a broom. Therefore, patients with semantic dementia that impairs ventral stream functions would be particularly impaired in recognizing and selecting MTTA based on their associated functions, as MTTA are functionally opaque. In contrast, due to substantial sensorimotor decoupling, modern tools (and even more so technological assistants) could support tool use in patients with apraxia despite their sensorimotor deficits.

From an ontogenetic or evolutionary perspective, it is an open question whether classical tool mastering is a prerequisite for MTTA mastering. For instance, does manipulation knowledge need to be acquired before being able to use increasingly sensorimotor decoupled MTTA, or does the latter even allow for tool use with less derived cognitive and manual prerequisites? On the one hand, primates are quite able to use human touchscreens (Egelkamp and Ross, 2019), e.g., to navigate an avatar through three-dimensional space (Allritz et al., 2022). On the other hand, young children have difficulties in transferring tool use strategies learned from 2D screen media to real-life (Moser et al., 2015; Hipp et al., 2017). Moreover, a pre-disposition for social learning – acquired during ontogenesis or phylogenesis – and perhaps even specific social learning mechanisms (e.g., imitation learning, supervised learning) may be required to master some of the MTTA as their design often does not point to their functions and proper use. Thus, the ability to follow verbal explanations and social instruction may become increasingly relevant when the function of a tool becomes more opaque (Needham, 2016; Zosh et al., 2017). Because perceivable functional properties are crucial for transferring functional tool-selection abilities to novel objects, another open question is how primates and children would do this for MTTA (e.g., a touchscreen or joystick), and which species-specific competencies (e.g., language, social-cognitive abilities) and learning strategies would be involved. At the neurofunctional level, learning to use novel tools or to substitute tools is accompanied by a dynamic learning curve from dorsal to ventral representations (Tobia and Madan, 2017). Thus, MTTA as compared to classical tools call for different competencies, or an adapted use of the tool-related basic faculties described here.

Lastly, tool use in VR adds an exciting methodological approach to analyze the entire complexity of tool mastering. During the user’s movements with a placeholder object, VR allows for simulating the tool, the target object, and the interaction between simulated tool and target object. Such simulations provide a unique opportunity to investigate the postulated basic faculties of tool mastering in an extraordinarily controlled manner. For instance, perceptual tool properties and sensorimotor feedback about tool use could be arbitrarily and gradually modulated and tested (Candidi et al., 2017). Concerning the increased sensorimotor decoupling in MTTA, this could show which features are essential for the application of stored manipulation knowledge on these modified tools, or at which point these modifications would call for new learning strategies based on mechanical reasoning. VR could also further elucidate the interaction between ventral and dorsal streams by manipulating the effect of tool-related actions on the perceivable state of the simulated tools. Moreover, dorso-ventral interaction during tool (use) processing may vary as a function of the level of sensorimotor decoupling. In neurological patients, VR tool use has been found to be beneficial in improving upper limb function when employed as an adjunct to usual care (Ke et al., 2017). However, only a few studies with neurological patients have investigated tool use in VR or the effects of modulating either manipulation knowledge or functional knowledge related to MTTAs (Candidi et al., 2017). Importantly, virtual tool use can be conceived of as both, an experimental technique to disentangle perceptual, motor, and cognitive components of tool mastering, but also as an object of research on its own.

We assume that our conceptual framework with the three basic faculties of tool mastering will be particularly beneficial for research that aims at uncovering the complex functional architecture of mastering modern tools. An interdisciplinary approach seems indispensable to study the specific sensorimotor and cognitive capacities, the types of learning involved, the age- or species-related limitations, or impairments in specific populations such as neuropsychological patients. This will transfer methods, evidence, and theoretical models gained in the past to the world of MTTAs.

## 6. Future perspectives

What kind of functional cognitive architecture is required for an autonomous system – living or artificial – that uses learning and self-organization to master classical and modern tools or technological assistants? In this paper, we have proposed a conceptual framework that seeks to motivate, facilitate, and structure interdisciplinary research on this core question. In particular, we advertise that tool mastering results from the interplay and integration of tool-related manipulation knowledge (combining tool perception and handling), functional knowledge (combining tool perception and selection), and means-end knowledge (combining tool handling and selection). The framework may also stimulate new interdisciplinary research, as it reveals that many important questions remain open, especially regarding the precise capabilities that enable tool mastering, their ontogenetic and phylogenetic development, differential relevance, and concerted integration required.

In the previous sections, we have also carved out how and why interdisciplinary approaches will enable or deepen our understanding of the basics and emergence of competent (human) tool mastering. To mention only some examples, future research may address the sensorimotor brain activity that accompanies young children’s object-directed actions (Yoo et al., 2016), developmental robotics aims at modelling ontogenetic sensorimotor development (Cangelosi and Schlesinger, 2018), and researchers may more systematically compare the limitations and abilities of tool mastering in children and non-human species (Lockman and Kahrs, 2017; Colbourne et al., 2021). Together with archaeological findings of tools we learn more about human evolution (Stout and Chaminade, 2012; Haslam et al., 2017). Further unsolved questions relate to the implicit and explicit learning of tool use skills in neurological patients and how the learning strategies adopted by patients compare to those used by children or non-human primates (Jacobs et al., 1999; Dovern et al., 2011).

Moreover, we think that comparisons between mastering classical and modern tools (and technological assistants), as well as studying tool use in VR, will provide unique windows to scrutinize the functional cognitive architecture of tool mastering. The increased sensorimotor decoupling and functional opacity of MTTA query the particular roles of sensorimotor feedback, manipulation knowledge, and means-end knowledge. At the same time, MTTA and tool use in VR enable an individual to pursue truly novel goals, because they enormously boost the tool user’s abilities to change the conditions of other objects or entities, with fewer demands on specific perceptual abilities and motor skills. Therefore, future studies should address the development and acquisition of MTTA mastering in children, or the limitations or impairment of tool mastering in older persons, neurological patients, or non-human primates, thereby considering that MTTA can enable tool use in individuals who (for various reasons) struggle with classical tool mastering.

Finally, experimental investigations should culminate in, and be supported by, computational modelling. As discussed above, most computational approaches do not exceed the complexity of inverse- or forward-models trying to capture the cause-effect properties of tool use. This leads to the fact that for robotic systems, functional tool-use knowledge is either imposed (programmed in) by the designer of the systems or, for more autonomous, self-learning robots, we observe still only a very rudimentary functional knowledge similar to that of young children and some non-human primates. This highlights that we need more solid computational foundations for tool mastering and robotics-based tests of such theories.

## Author contributions

All authors listed have made a substantial, direct, and intellectual contribution to the work, and approved it for publication.

## Funding

We gratefully acknowledge the funding of a roundtable discussion on “Tool Choosing and Tool Using” by the German Research Foundation (DFG; SCHU 1439/9-1). All authors are funded by the German Research Foundation (DFG): RS: Project-IDs 397530566 and 515997569. SE: Project-ID 454726751. BE: Project ID 277140543 - FOR 2253. PW: Project-ID 431549029 - SFB 1451. FW: collaborative research center SFB 1528, sub-project B01.

## Conflict of interest

The authors declare that the research was conducted in the absence of any commercial or financial relationships that could be construed as a potential conflict of interest.

## Publisher’s note

All claims expressed in this article are solely those of the authors and do not necessarily represent those of their affiliated organizations, or those of the publisher, the editors and the reviewers. Any product that may be evaluated in this article, or claim that may be made by its manufacturer, is not guaranteed or endorsed by the publisher.
